# *EYS* mutations and implementation of minigene assay for variant classification in EYS-associated retinitis pigmentosa in northern Sweden

**DOI:** 10.1038/s41598-021-87224-9

**Published:** 2021-04-08

**Authors:** Ida Maria Westin, Frida Jonsson, Lennart Österman, Monica Holmberg, Marie Burstedt, Irina Golovleva

**Affiliations:** 1 Medical Biosciences/Medical and Clinical Genetics, University of Umeå, 901 87 Umeå, Sweden; 2 Clinical Science/Ophthalmology, University of Umeå, 901 85 Umeå, Sweden

**Keywords:** Genetics, Medical research

## Abstract

Retinitis pigmentosa (RP) is a clinically and genetically heterogeneous group of inherited retinal degenerations. The ortholog of Drosophila eyes shut/spacemaker, *EYS* on chromosome 6q12 is a major genetic cause of recessive RP worldwide, with prevalence of 5 to 30%. In this study, by using targeted NGS, MLPA and Sanger sequencing we uncovered the *EYS* gene as one of the most common genetic cause of autosomal recessive RP in northern Sweden accounting for at least 16%. The most frequent pathogenic variant was c.8648_8655del that in some patients was identified in *cis* with c.1155T>A, indicating Finnish ancestry. We also showed that two novel *EYS* variants, c.2992_2992+6delinsTG and c.3877+1G>A caused exon skipping in human embryonic kidney cells, HEK293T and in retinal pigment epithelium cells, ARPE-19 demonstrating that in vitro minigene assay is a straightforward tool for the analysis of intronic variants. We conclude, that whenever it is possible, functional testing is of great value for classification of intronic *EYS* variants and the following molecular testing of family members, their genetic counselling, and inclusion of RP patients to future treatment studies.

## Introduction

Retinitis pigmentosa (RP, MIM 268000) is a clinically and genetically heterogeneous group of inherited retinal degenerations (IRDs) causing primarily deterioration of the rod photoreceptors with subsequent worsening of the cones. It is classified into two forms: syndromic (affecting other organs) and non-syndromic (not affecting other organs). The initial symptom of RP is night blindness followed by decreased peripheral vision and progression to legal blindness^1^. The prevalence worldwide is estimated to 1/4000^2–4^ but in isolated regions it is 1/1000–1/2000 due to founder effects^5–7^.

RP can be inherited in autosomal dominant (ad, 30–40%), autosomal recessive (ar, 50–60%) and X-linked mode (5–15%)^8^. RP cases with digenic inheritance have also been reported but are very rare^9^. To date, according to the Retinal Information Network (https://sph.uth.edu/retnet/) 65 genes associated with non-syndromic RP are identified, however, majority of them cause the disease in 1–2% of all cases.

One of the genes, the ortholog of Drosophila eyes shut/spacemaker, *EYS* (MIM #612424) causing autosomal recessive RP (arRP) in humans was discovered in 2008^10,11^. The gene resides on chromosome 6q12 and was originally denoted as RP25 locus. Since then, *EYS* was shown to be a major gene for recessive RP worldwide, with prevalence 5–30%^12–17^. There is a large diversity in spectrum of *EYS* mutations. Analysis of 205 known *EYS* mutations in McGuigan’s study showed that 49% are truncating mutations, 37% are missense mutations, 8% are large deletions affecting one or more exons and 6% are splice-sites mutations^18^. Recent analysis of the 297 unique *EYS* variants also demonstrated that 44% are predicted to result in premature truncation of the *EYS* protein^19^. Nowadays, gene variants are classified based on the American College of Medical Genetics and Genomics (ACMG) guidelines^20^ and some *EYS* variants previously reported pathogenic have been re-classified as variants of uncertain significance^19^. The variant re-classification highlights the need of functional assays to understand the role of *EYS* variants in the pathogenic mechanisms underlying EYS-associated RP. The fact that *EYS* is exclusively expressed in retina and is absent in several rodent species, makes it difficult to generate animal models of the disease^10,19^. Furthermore, functional effect of the variants such as missense and nonsense can be predicted by computational tools, however variants in non-coding regions e.g., introns are more difficult to assess regarding their impact on protein function. Therefore, functional analysis of non-coding mutations might be critical in some cases.

In this study, we explored a cohort of RP patients (n = 81) that included 65 arRP cases with unknown genetic cause of their disease and aimed to investigate frequency of *EYS* mutations using cascade-targeted mutation analysis. We showed that *EYS* gene is a common cause of arRP in northern Sweden and some of the Swedish patients share an *EYS* founder allele present in Finnish population. We also illustrated benefits of a minigene assay for demonstration of effects of intronic variants on splicing of the *EYS* gene in human embryonic kidney cells, HEK293T and in retinal pigment epithelium cells, ARPE-19.

## Results

### *EYS* mutations in the RP cohort from northern Sweden

81 cases with clinical diagnosis of RP were available for the mutation analysis. The inheritance pattern was defined from the family history but remained uncertain in some cases. DNA from patients with arRP, RP18, RP26, RP74, RP103, RP154, RP160, RP165, RP6795 and VC101 (Table 1, Supplementary Figure S2) was analysed using a targeted analysis of 501 known variants in 16 arRP-associated genes, *EYS* not included. No mutations were identified by that time. Later, RP160 (Family 501, Supplementary Figure S1, Figure S2) analysed by NGS with a 56 arRP-associated gene panel, was found to be heterozygous for a novel sequence variant; an 8 bp deletion in exon 43 in the *EYS* gene c.8648_8655del. Two other affected family members (RP5436 and RP6795) were also heterozygous carriers of the same deletion. Sanger sequencing of exon 43 in 78 RP patients revealed five heterozygotes (RP15, RP18, RP74, RP103 and RP105) and five homozygotes (VC101, RP26, RP154, RP165 and RP6019) (Table 1). As a next step, all coding sequences of the *EYS* gene were sequenced in the eight heterozygotes to identify a second pathogenic variant (RP15, RP18, RP74, RP103, RP105, RP160, RP5436 and RP6795). Three potential culprit variants were detected c.1155T>A (RP15, RP103, RP105), c.2992_2992+6delinsTG (RP15) and c.3877+1G>A (RP103 and RP105) (Table 1, Supplementary Figure S3). Consequently, all 81 RP patients were sequenced regarding the three identified *EYS* variants. The c.1155T>A was found in *cis* with the 8 bp deletion (c.8648-8655del) in four patients from Family 012 (VC101, RP15) and Family 335 (RP103, RP105). All four patients were carriers of bi-allelic mutations; VC101 was homozygous for the *cis* allele, RP15 was a compound heterozygote with c.2992_2992+6delinsTG, RP103 and RP105 were compound heterozygotes with 3877+1G>A (Table 1). The five remaining RP cases (RP18, RP74, RP160, RP5436 and RP6795) with only one *EYS* mutation were subjected to MLPA to detect copy number variants. By using a MLPA probe ligation site situated 133 bp before exon 42 we detected a ~ 41 kb duplication of exon 42 in three members of Family 501. The size of the duplication was narrowed to a ~ 13 kb area between genomic coordinates chr6:63,721,660 in exon 43 and chr6:63,734,700 in intron 41 (Genome Browser GRChr38/hg38) by using of three custom designed MLPA probes (Supplementary Figure S4). The exact breakpoints of the duplication could not be identified. Three affected individuals in the Family 501 were shown to be compound heterozygotes inheriting the duplication and the 8-bp deletion from each parent. The second *EYS* mutation in RP18 and RP74 remains unknown. Thus, our cascade-targeted mutation analysis allowed identification of *EYS* mutations in 14 of 81 RP cases. Next, we sequenced all coding regions of the *EYS* gene and performed MLPA in additional 24 of the 65 arRP cases. The only noteworthy finding was detection of a previously reported variant c.6284C>T, p.(Pro2095Leu) in patient RP85. Other variants were deemed benign based on computational and mining resources. No large duplications or deletions were detected by MLPA. Summary of the diseases associated *EYS* variants detected in this study is presented in Table 2 and Supplementary Figure S3.

**Table 1:** *EYS* variants in arRP patients in the northern part of Sweden.

Family	Sample	Age	Origin	Allele 1	Allele 2	Zygosity	Protein change
012	RP15	92 y.o	Västerbotten	c.[1155T>A;8648_8655del]	c.2992_2992+6delinsTG	hetero	p.[(Cys385*, Thr2883Lysfs*4)];[(Phe950Hisfs*4)]^**1**^
012	VC101	87 y.o	Västerbotten	c.[1155T>A;8648_8655del]	c.[1155T>A;8648_8655del]	homo	p.[(Cys385*, Thr2883Lysfs*4)];[(Cys385*, Thr2883Lysfs*4)]^**2**^
335	RP103	85 y.o	Västernorrland	c.[1155T>A;8648_8655del]	c.3877+1G>A	hetero	p.[(Cys385*, Thr2883Lysfs*4)];[(Thr1229Valfs*21)]^**1**^
335	RP105	90 y.o	Västernorrland	c.[1155T>A;8648_8655del]	c.3877+1G>A	hetero	p.[(Cys385*, Thr2883Lysfs*4)];[(Thr1229Valfs*21)]^**1**^
501	RP160	32 y.o	Norrbotten	c.8648_8655del	g.(63721660_63723982)_(63728011_63734700)dup	hetero	p.[(Thr2883Lysfs*4)];[?]^**3**^
501	RP5436	19 y.o	Norrbotten	c.8648_8655del	g.(63721660_63723982)_(63728011_63734700)dup	hetero	p.[(Thr2883Lysfs*4)];[?]^**3**^
501	RP6795	17 y.o	Norrbotten	c.8648_8655del	g.(63721660_63723982)_(63728011_63734700)dup	hetero	p.[(Thr2883Lysfs*4)];[?]^**3**^
simplex	RP26	65 y.o	Norrbotten	c.8648_8655del	c.8648_8655del	homo	p.[(Thr2883Lysfs*4)];[(Thr2883Lysfs*4)]^**2**^
simplex	RP85	62 y.o	Norrbotten	c.6284C>T	?	hetero	p.[(Pro2095Leu)];[?]^**4**^
simplex	RD154	49 y.o	Västerbotten	c.8648_8655del	c.8648_8655del	homo	p.[(Thr2883Lysfs*4)];[(Thr2883Lysfs*4)]^**2**^
simplex	RP165	59 y.o	Västerbotten	c.8648_8655del	c.8648_8655del	homo	p.[(Thr2883Lysfs*4)];[(Thr2883Lysfs*4)]^**2**^
simplex	RP6019	57 y.o	Norrbotten	c.8648_8655del	c.8648_8655del	homo	p.[(Thr2883Lysfs*4)];[(Thr2883Lysfs*4)]^**2**^
simplex	RP18	81 y.o	Norrbotten	c.8648_8655del	?	hetero	p.[(Thr2883Lysfs*4)];[?]^**4**^
simplex	RP74	77 y.o	Norrbotten	c.8648_8655del	?	hetero	p.[(Thr2883Lysfs*4)];[?]^**4**^

^**1**^Two variants are present on one allele and another variant is present on the second allele in this patient.

^**2**^Pathogenic variants were present on both alleles in this patient.

^**3**^Duplication breakpoints remain unknown.

^**4**^Only one likely pathogenic variant was found. All sequence positions are denoted according to NM_001142800.1 (human reference genome GRCh38/hg38).

**Table 2:** Characterization of *EYS* variants identified in arRP cohort from northern Sweden.

cDNA	Genomic position (start) GRCh38	dbSNP	gnomAD Exomes/Genomes	DANN/CADD/ MutationTaster	GERP/RS	FATHMM-MKL	ACMG pathogenicity criteria
c.1155T>A	chr6:65,402,507	rs143994166	0.000727/0.001	0.9831/34/disease-causing	− 0.8669	Neutral	PVS1, PM2, PP5
c.2992_2992+6delinsTG	chr6:64,886,691	–	0/0	–	2.24	–	PVS1, PM2, PP3
c.3877+1G>A	chr6:64,593,116	–	0/0	0.8183/22.4/polymorphism	3.64	Neutral	PVS1, PM2, PP3
c.6284C>T	chr6:64,230,732	rs200374024	0.000541/0.000511	0.9925/16.4/polymorphism	3.58/0.73	Neutral	BP1, BP4
c.8648_8655del	chr6:63,721,376	rs528919874	0.00079/0.000905	–	3.39	–	PVS1, PM2, PP5
dup exon 42	chr6:63,723,984	–	-	–	–	–	–

All sequence positions are denoted according to NM_001142800.1) (GRCh38/hg38 human reference genome).

dbSNP –database of single nucleotide variations at https://www.ncbi.nlm.nih.gov/snp/. gnomAD-genome aggregation database at https://gnomad.broadinstitute.org/.

DANN/CADD-prediction tools for annotating of pathogenicity (Quang et al. 2015; Rentzsch et al. 2019) MutationTaster predicts a disease-causing potential of single nucleotide polymorphism (Schwarz et al. 2014). GERP-Genomic Evolutionary Rate Profiling (GERP) is a conservation score across alignments of the genomes of 35 mammals (Cooper et al. 2005). FATHMM-MKL represents normalized functional predictions from numerous non-synonymous computational predictions algorithms (Shihab et al. 2017). ACMG- standards and guidelines for the interpretation of sequence variants recommended by the American College of Medical Genetics and Genomics (Richards et al. 2015).

### In silico prediction of the c.2992_2992+6delinsTG and c.3877+1G>A variants on splicing

To evaluate association of the two *EYS* intronic variants with arRP and their role in gene splicing, we first performed in silico analysis. The effect of splice-site variants was calculated by the algorithms included in Alamut Visual software version 2. Both variants were classified as pathogenic according to ACMG. The c.2992_2992+6delinsTG variant in intron 19 of the *EYS* gene was predicted to alter the donor site at a risk of 100% by MaxEnt, 100% by NNSPLICE and 100% by HSF, giving an average risk prediction of aberrant splicing of 100%. The canonical c.3877+1G>A variant in intron 25 of the *EYS* gene was also predicted to alter the donor site 1 bp upstream of the mutation at a risk of 100% by MaxEnt, 100% risk by NNSplice and 100% risk by HSF, giving an average risk prediction of aberrant splicing of 100%.

### In vitro splice assay using *EYS* minigenes carrying c.2992_2992+6delinsTG variant

**Figure 1:**
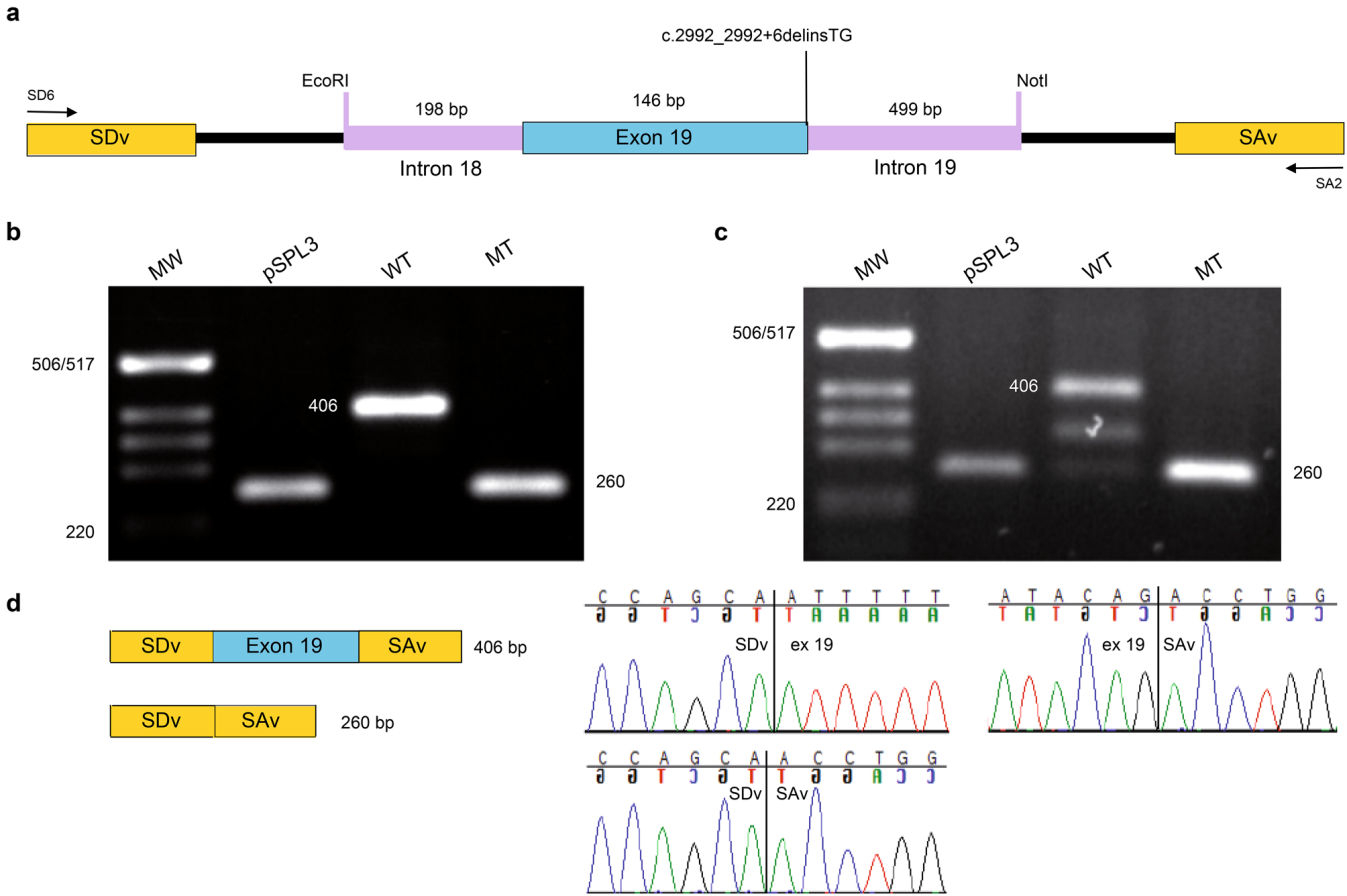
Exon skipping because of *EYS* c.2992_2992+6delinsTG variant. (**a**) A schematic illustration of the pSPL3-*EYS* c.2992_2992+6delinsTG minigene. Exon 19 of *EYS* gene (blue) and flanking introns (purple) were cloned into the pSPL3 vector (black) with a wildtype (wt) or mutant (mt) c.2992_2992+6delinsTG between two pSPL3 exons (yellow); splice donor vector (SDv) and splice acceptor vector (SAv). Positions of SD6 and SA2 primers for splice analysis are displayed. HEK293T cells and ARPE-19 cells were transfected with wt or mt *EYS* hybrid minigene or an empty pSPL3 vector. After RNA extraction and cDNA synthesis, splicing products were amplified by PCR using SD6 and SA2 vector specific primers and visualized by agarose gel electrophoresis. Resolution of splicing PCR products derived from HEK293T are shown in (**b)** and from ARPE-19 in (**c)**. Sanger sequencing confirmed that in the control pSPL3, only SDv-SAv splicing occurred, leading to a 260 bp product. Expected wt splicing of the *EYS* minigene was a 406 bp product. In ARPE-19 cells, an additional band ~ 320 bp of unknown sequence was visible for the wt. In both HEK293T and ARPE-19 cells, the mt hybrid minigene resulted in exon skipping (**d**). Full-length gels are presented in Supplementary Figure S7.

To analyse how the *EYS* c.2992_2992 + 6delinsTG variant affects splicing, wt and mt *EYS* minigenes covering exon 19 and part of the adjacent intron sequence were generated (Fig. 1). The minigenes were transfected into HEK293T and ARPE-19 cells and the splicing was analysed. The wt *EYS* minigene transfected into HEK293T cells resulted in one distinct product of 406 bp (Fig. 1). By sequencing, we could confirm that the 406 bp product represents correct splicing of exon 19. Transfection of the mt *EYS* c.2992_2992+6delinsTG minigene into HEK293T cells resulted in a 260 bp product, due to exon 19 skipping (Fig. 1). Transfection of the ARPE-19 cells with wt *EYS* minigene resulted in three products; 406 bps, ~ 320 bps and 260 bps (Fig. 1), where correctly spliced transcript was the most prevalent. When the mt *EYS* c.2992_2992+6delinsTG minigene was transfected into ARPE-19 only the product with skipped exon 19 was observed (Fig. 1). Thus, we observed exon skipping in both human embryonic kidney HEK293T and retinal pigment epithelium ARPE-19 cells when the c.2992_2992+6delinsTG variant was introduced. Skipping of exon 19 is predicted to result in a premature stop codon and due to absence of exon 19, the amino acid nomenclature would be p.(Phe950Hisfs*4).

### In vitro splice assay using *EYS* minigenes with the c.3877+1G>A variant

**Figure 2:**
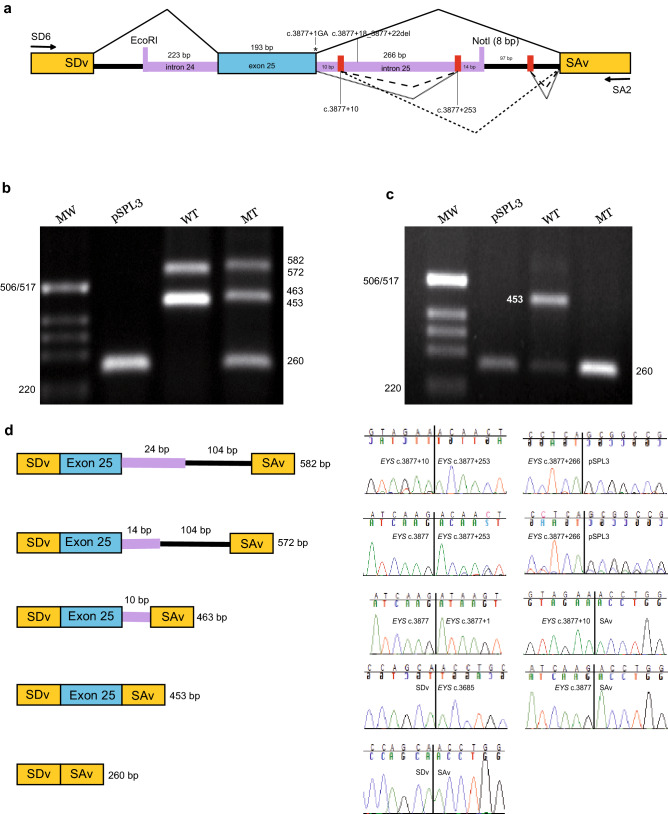
Exon skipping because of *EYS* c.3877+1G>A variant. (**a**) A schematic illustration of the pSPL3-*EYS* c.3877+1G>A minigene. Exon 25 of *EYS* gene (blue) and flanking introns (purple) were cloned into the NotI and EcoRI sites of pSPL3 vector (black) with a wildtype (wt) or mutant (mt) c.3877+1G>A between two pSPL3 exons (yellow); splice donor vector (SDv) and splice acceptor vector (SAv). Positions of SD6 and SA2 primers for splice analysis are displayed. Red blocks show two alternative splice sites in intron 25 and one alternative splice site in pSPL3. In the patient’s DNA used for minigene construction, a common deletion at position c.3877+18_3877+22del on the same allele as c.3877+1G>A was present. Solid and dashed lines show different outcomes of splicing. HEK293T cells and ARPE-19 cells were transfected with wt or mt *EYS* hybrid minigene or an empty pSPL3 vector. After RNA extraction and cDNA synthesis, splicing products were amplified by PCR using SD6 and SA2 vector specific primers and visualized by agarose gel electrophoresis. Resolution of splicing PCR products derived from HEK293T are shown in (**b**) and from ARPE-19 in (**c**). Sanger sequencing confirmed that in the control pSPL3, only SDv-SAv splicing occurred, leading to a 260 bp product. Expected wt splicing of the *EYS* minigene was a 453 bp product. Additional splicing took place in the wt minigene transfected into HEK293T cells where incorporation of 14 bp of the 3′ end of the *EYS* amplicon occurred, starting at c.3877+253 (red block) and continuing 97 bp into the pSPL3 vector before splicing to SAv. This aberrant splicing resulted in a 572 bp product. The same incorporation occurred in the mt minigene in HEK293T, with an additional 10 bp of consecutive intron 25 sequence in the 5′ end, leading to a 582 bp product. In HEK293T, mt minigene also produced an mRNA with expected splicing at the SDv-exon 25 junction, but with the incorporation of 10 bp consecutive sequence of intron 25, before joining SAv. This was not seen in ARPE-cells. In both HEK293T and ARPE-19 cells, the mt hybrid minigene resulted in exon skipping (**d**). Full-length gels are presented in Supplementary Figure S7.

To demonstrate how the *EYS* c.3877+1G>A variant affects splicing, wt and mt *EYS* minigenes were generated (Fig. 2). The minigenes were transfected into HEK293T and ARPE-19 cells and the splicing outcome was analysed. The wt *EYS* minigene transfected into HEK293T cells resulted in two distinct products of 572 bp and 453 bp respectively (Fig. 2). The 453 bp product corresponded to correct splicing of exon 25, while the 572 bp fragment was the result of using exon 25 donor site and pSPL3 alternative donor site. The sequencing revealed inclusion of 119 nucleotides that covered *EYS* intron 25 sequence from c.3877+253 to c.3877+266 (14 bp) plus the entire NotI restriction site (8 bp), and continuing 97 bp into the pSPL3 vector before splicing out at a cryptic donor site within the pSPL3, and thereafter joining pSPL3 acceptor site SAv.

Transfection of the mt *EYS* c.3877+1G>A minigene into HEK293T resulted in three products: 582 bp, 463 bp and 260 bp (Fig. 2). The 582 bp fragment had additional 10 bp of intron 25 sequence (c.3877+10) and 119 nucleotides as seen in the wt construct. The 463 bp product was shown to include the same 10 bp of intron 25 sequence as seen in the 582 bp product. No correctly spliced product was observed with the mt *EYS* c.3877+1G>A minigene. The 260 bp fragment was the result of exon 25 skipping. Transfection of the ARPE-19 cells with wt *EYS* minigene resulted in two products: 453 bps and 260 bps (Fig. 2), with the majority representing the correctly spliced transcript. When the mt *EYS* c.3877+1G>A minigene was transfected into ARPE-19 only the product with exon 25 skipping was observed (Fig. 2). Thus, exon skipping was demonstrated in both human embryonic kidney HEK293T and retinal pigment epithelium ARPE-19 cells when the c.3877+1A variant was introduced. Skipping of exon 25 would result in an early stop codon and due to absence of exon 25, the amino acid nomenclature would be p.(Thr1229Valfs*21).

### Clinical findings

**Figure 3:**
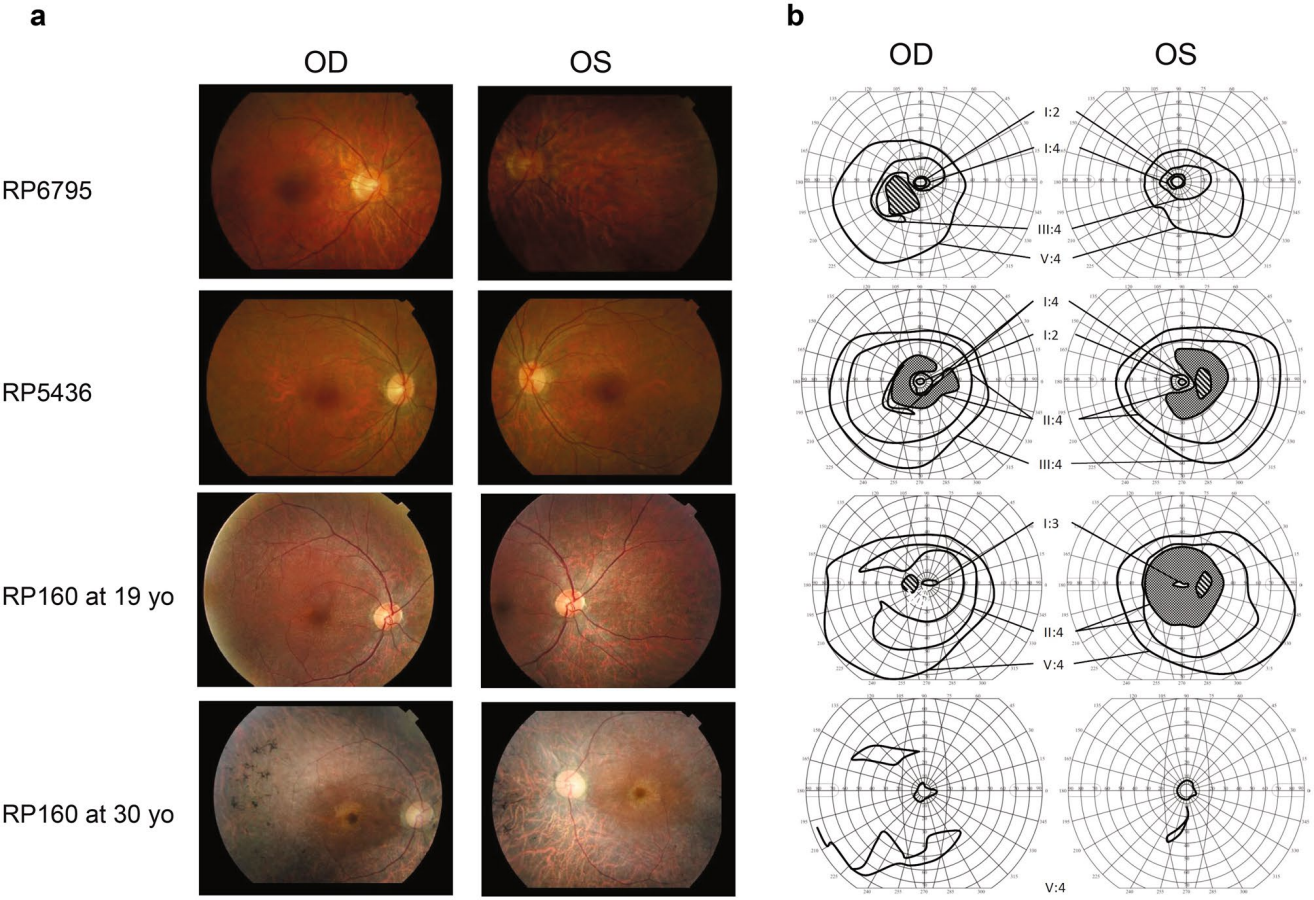
Fundus photographs (**a**) and visual fields (**b**) in affected family members RP6795, female, examined at 15 yo; RP5436, female, examined at 18 yo; RP160, male, examined at 19 and 30 yo. Paracentral scotomas are seen in RP6795, and RP5436. Absolute paracentral scotomas were seen in RP160 at 19 yo that advanced to minor central/peripheral islets when re-examined at 30 yo. Absolute scotomas are shown as stripes and relative scotomas are shown as checkered.

In this study we could only investigate clinical presentation of *EYS*-associated RP in three individuals from Family 501 (Table 3). Visual acuity was unaffected with no significant cataract or other ophthalmologic diseases present during the first two decades for all three patients. On fundoscopy, normal optic discs, discrete arteriolar attenuation and single pigmentary deposits in the periphery were seen in RP6795 at 15 yo, in RP5436 at 18 yo and in RP160 at 19 yo (Fig. 3). Upon visual field examinations, case RP6795 had restricted peripheral visual fields with relative paracentral scotoma (Fig. 3). Her younger female relative, RP5436, had less affected peripheral vision with arcuate relative scotomas in the mid-periphery. RP160 at 19 yo presented a large paracentral scotoma on both eyes (Fig. 3). OCT performed in RP5436 and RP160 demonstrated a general thinning of the macula with some foveal sparing (Supplementary Figure S5). The full-field standard dark-adapted ERGs are presented in Supplementary Figure S6. The amplitudes of rod, mixed rod-cone, cone and 30 Hz flicker responses were all subnormal to non-recordable in all cases at young age, meaning that the photoreceptor functions were seriously affected already in their teens. The young man, RP160, examined at age 19 and re-examined at age 30, presented several indicators of disease progression during this period. On fundoscopy the optic discs had gained a waxy pallor, alongside severe arteriolar attenuation, and an indication of a bull-eye lesion in the macula (Fig. 3). The retina was generally atrophic with the typical bone spicule pigmentation. His VF had severely deteriorated with some preserved central and peripheral islets. There was a notable progression in the visual field loss over time, going from arcuate and ring-shaped scotomas to only mid-central preservation (Fig. 3). ERG showed a decline of response amplitudes over time for both rods and cones, and the rod, mixed rod/cone responses were extinguished at age 30 (Supplementary Figure S6).

**Table 3:** Clinical characterization of patients with *EYS* associated RP in family 501.

Case/sex/age	Onset age of NB	VA LogMAR OD/OS	Refractive errors OD/OS	Photophobia
RP6795/F/15	12	0.00/− 0.20	+ 0.4/ + 0.25	−
RP5436/F/18	18	0.00/0.10	+ 0.4/ + 0.5	+
RP160/M/19	15	0.20/0.10	− 2.0/ − 0.75	+
RP160/M/30	15	0.50/0.30	− 1.5/ − 1.0	+

M male, F female, NB night blindness, VA visual acuity, OD right eye, OS left eye.

+ sign = present; − sign = not present.

## Discussion

Taken into account a relative homogeneity of the population in northern Sweden and presence of founder and family specific mutations in several retinal genes^5,21–27^ we were looking for shared genetic causes in genetically unresolved cases of arRP in northern Sweden. In this study we investigated RP cohort including 65 arRP patients with unknown genetic cause and identified mutations in the *EYS* gene in 14 cases including 4 family members. Thus, the *EYS* mutations were present in 16% of arRP cases.

Two of six *EYS* mutations detected in this study were reported previously, c.1155 T > A in exon 7^18,28^ and c.8648_8655del in exon 43^12,19^. Both mutations present on the same allele had been found earlier in three Finnish patients with retinal dystrophy^29^. The most common *EYS* variant in our study, c.8648_8655del was present in 13 cases. We were not able to connect the families of all 13 carriers but considering that 6 patients originate from the same region of Tornedalen (Pajala and Karesuando) adjacent to the Finnish boarder, it is likely that they have a common ancestor. Four of the 13 patients carried c.8648_8655del and c.1155T>A variants in *cis* indicating that they share the same ancestor as the Finnish cases^29^. The Finnish origin of the *cis* allele might explain why we were not able to relate the Swedish families using the Swedish church records.

In two patients, RP18 and RP74 we found only one likely pathogenic variant c.8648_8655del. As we only investigated the intronic sequences adjacent to the *EYS* exons, a deep intronic variant as a second causal variant cannot be ruled out. The same could be true for RP85 where we found only one heterozygous missense variant, c.6284C>T reported once in a compound heterozygous arRP patient with second *EYS* variant c.2234A>G^30^. Both missense variants are classified as variants with uncertain significance in ClinVar (https://www.ncbi.nlm.nih.gov/clinvar/) and no functional studies have been done on either of the variants. Therefore, the pathogenicity of c.6284C>T is questionable.

A large, likely pathogenic, duplication covering an approximately 13 kb region between chr6:63,721,660 in exon 43 (c.8381) and chr6:63,734,700 (c.8072–8019) in intron 41 was identified in Family 501. However, due to the lack of additional MLPA probes we could not reveal the breakpoints of the aberration covering exon 42, as Pieras et al*.* could not do for the duplication of exons 3 and 4 in the *EYS* gene^31^. In our cohort we detected genomic rearrangements in only one family, compare to 4% *EYS* CNV in French cohort^31^.

Since the pathogenic mechanisms in EYS-associated IRD are still poorly understood, the interpretation of the pathogenicity of many *EYS* variants is a challenge. The implementation of numerous computational tools to predict functionality of potentially disease-causing variants underscores the need of functional assays especially for the variants outside coding regions. According to Messchaert et al. (2018) the large size of *EYS’* cDNA, the absence in the genome of mice and rats and the retina specific expression represent the factors limiting an experimental assessment of the pathogenicity of *EYS* variants^19^. In this study we assessed functionality of two intronic variants both predicted to be pathogenic due to the changes in exon–intron boundaries. By implementation of in vitro minigene assays we easily confirmed exon skipping caused by both *EYS* variants, c.2992_2992+6delinsTG and c.3877+1G>A. It is worth to mention that splicing outcome of in vitro minigene assays depends on the content of DNA sequences of the gene of interest and the cells chosen for the assay. We found that an inclusion of 10 bp of consecutive intron 25 occurred in HEK293T cells transfected with mutant minigene *EYS* c.3877+1G>A, but not in ARPE-19 cells. Computational tools revealed two alternative sites in intron 25, 10 and 14 bp from exon–intron junction site. In the patient’s DNA from which the minigene was constructed a common deletion at position c.3877+18_3877+22del on the same allele as c.3877+1G>A was present. This 5 bp deletion was predicted to quench the stronger alternative donor site at position + 14, corroborating our result. Not only mutant but also wildtype minigene showed complex splicing by incorporating a part of the amplicon’s intron sequence at position c.3877+253 and retaining 97 bp of the pSPL3. This aberrant splicing is more likely to be HEK293T specific because it was not seen in the ARPE-19 cells.

Mutations in the *EYS* gene cause a variety of phenotypes such as RP and cone-rod dystrophy characterised by inter- and intrafamilial phenotypic diversities^10,13,32,33^. We provide clinical evaluation of only three patients from one family available for this study.

The proposed molecular mechanism of RP caused by *EYS* mutations is loss of protein function. The role of EYS is not yet understood but presence of epidermal growth factor-like and laminin domains might be important in cell adhesion, migration and intracellular signalling^34,35^. Also, it is known that an ortholog of the Drosophila spacemaker (spam) protein plays a critical role in maintenance of the photoreceptor morphology^36^. Currently 343 *EYS* mutations are reported in The Human Gene Mutation Database (http://www.hgmd.cf.ac.uk/) with almost 44% predicted to result in protein truncation^19^. Nonsense or protein truncating variants are usually easy to classify as pathogenic according to ACMG recommendations^20^ however missense and intronic variants require additional factors to be classified as likely pathogenic or pathogenic. According to the last *EYS* update^19^ missense and splice site *EYS* variants represent 48.2% of all mutations, but only 8.6% can be classified as likely pathogenic while the rest are variants of uncertain significance. Lack of functional studies prevent classification of the variants as pathogenic and, thus might dismiss these variants in future genetic testing and counselling.

Many defective transcripts are degraded by nonsense-mediated mRNA decay (NMD)^37^ that could also partially explain the phenotypic variation^38,39^. Recent studies on three *EYS* transcripts with pathogenic mutations c.1211dupA, c.4957dupA and c.8805C>A revealed barely detected, low and almost normal level of expression of the mutant allele which suggested that almost complete NMD, partial NMD and escape from NMD took place^40^. Interestingly, transcripts carrying the c.8805C>A mutation, that result in a premature termination codon in the last exon 43, escaped degradation by NMD and demonstrated normal expression levels^40^. The most common mutation in northern Sweden, c.8648_8655del and a large duplication located in exon 42 set grounds for study of a genotype–phenotype correlation.

A debatable pitfall of our study is the cascade-targeted mutation analysis of the *EYS* gene. Not all arRP patients in our cohort were subjected to the analysis of the entire *EYS* gene making it possible that some mutations escaped detection. However, our aim was to find common *EYS* variants in the homogenous population of northern Sweden and the 8 bp deletion found in 13 of arRP cases proved the case. The additional screening of the entire coding regions of the *EYS* gene in 24 unresolved arRP resulted in only one finding of a variant of uncertain significance (c.6284C>T). This leaves us with 28 unresolved arRP cases that have only been screened for the variants found in this study. The probability of finding yet another common *EYS* variant among these patients seems to be low.

In conclusion, our study revealed one of the most common genetic cause of autosomal recessive retinitis pigmentosa in northern Sweden. *EYS* mutations accounted for 16% of arRP cases with the most frequent pathogenic variant c.8648_8655del. The same mutation in *cis* with c.1155T>A indicated presence of Finnish founder allele in Swedish patients. We also demonstrated that in vitro minigene assay is important applicable tool for the functional analysis of intronic variants. If the pathogenic mechanisms for these variants can be determined, it will be of a great value for variant classification, molecular testing, genetic counselling, and future treatment of patients with EYS-associated RP.

## Methods

### Patients

In this study we used DNA from 81 patients with clinical diagnosis of retinitis pigmentosa (RP). Supposedly, 9 patients had autosomal dominant RP (adRP), 65 had autosomal recessive RP (arRP) and 7 patients had either adRP or arRP. Among arRP patients, 7 belonged to three families (Families 012, 335 and 501, Supplementary Figure S1) and the rest were simplex. Peripheral blood samples were collected in EDTA tubes and DNA was extracted as described elsewhere. The assignment of inheritance pattern was based on a family history provided by the patients. However, some of the elderly patients did not have details about visual impairment of their siblings and parents and most of the cases were regarded as simplex. DNA from siblings or parents was available only in three families. The study was approved by the Swedish Ethical Review Authority (Etikprövningsmyndigheten) and conducted in accordance with ethical principles for medical research involving human subjects as stated in Declaration of Helsinki. An informed consent was obtained from all patients or their parents before inclusion to the study. During first examination of patient RP6795 an informed consent was obtained from her parents.

### Molecular genetic analyses

*The arrayed primer extension assay (APEX)* was performed via Asper Biogene (Tartu, Estonia) (https://www.asperbio.com/asper-ophthalmics). DNA was analysed in 2006–2007 using a panel for arRP-associated 501 known variants in 16 genes (*CERKL* Gene ID-ENSG00000188452, OMIM 608,381; *CNGA1* Gene ID-ENSG00000198515, OMIM 123,825; *CNGB1-*ENSG00000070729, OMIM 600,724; *MERTK* Gene ID-ENSG00000153208, OMIM 604,705; *PDE6A*–Gene ID ENSG00000132915, OMIM 180,071; *PDE6B*–Gene ID ENSG00000133256, OMIM 180,072; *PNR/NR2E3*–Gene ID ENSG00000278570, OMIM 604,485; *RDH12*–Gene ID ENSG00000139988, OMIM 608,830; *RGR-*Gene ID ENSG00000148604, OMIM 600,342; *RLBP1*–Gene ID ENSG00000140522, OMIM 180,090;*SAG*–Gene ID ENSG00000130561, OMIM 181,031; *TULP1*–Gene ID ENSG00000112041, OMIM 602,280; *CRB1*–Gene ID ENSG00000134376, OMIM 604,210;*RPE65*–Gene ID ENSG00000116745, OMIM 180,069; *USH2A*–Gene ID ENSG00000042781, OMIM 608,400; and *USH3A/CLRN1-*Gene ID ENSG00000163646, OMIM 606,397).

*Next generation sequencing (NGS)* of 56 arRP-associated genes was performed at Asper Biogene. Samples were analysed using the BWA Enrichment app in BaseSpace (Illumina). Alignment was performed with BWA and variants were called with GATK^41^. Mean coverage was 87 reads for the whole gene panel and mean coverage of *EYS* gene was 99 reads. Variants that had an alternative variant frequency less than 20% and/or had a call quality score lower than 20 were automatically filtered out. Confirmation of NGS findings and subsequent screening of *EYS* gene was done with *Sanger sequencing* using BigDye Terminator v3.1 Cycle Sequencing Kit (Applied Biosystems, Foster City, CA, USA). Primer sequences for the screening of the *EYS* gene are available upon request. The products of sequencing reactions were analysed on ABI 3500xL Dx Genetic Analyser (Applied Biosystems). Sequences were aligned and evaluated using the Sequencher software version 4.9 (Gene Codes Corporation, Ann Arbor, MI, USA). All changes were assigned according to the GenBank Reference Sequence Version FJ416331; GI: 212,675,237; Transcript Reference Sequence: NM_001142800.1 and described according the HGVS recommendations^42^.

For detection of copy number changes in exonic sequences of the *EYS* gene we used *Multiplex Dependent Probe Amplification (MLPA)* with SALSA MLPA Probe mix P328-A1-0811 or P328-A2-0217 lacking probes for exon 9 or exons 2, 7, 9 and 27, respectively (MRC Holland, Amsterdam, Netherlands). Additionally, three MLPA probes were designed aiming to detect breakpoints of the duplication detected in Family 501. The sequences of these MLPA probes are available in Supplementary Figure S4. The MLPA reactions were run on ABI 3500xL Dx Genetic Analyser (Applied Biosystems) and the data was evaluated in GeneMarker v.2.7.0 (SoftGenetics, State College, PA, USA) with deletions set to be detected at quote values below 0.75 and duplications set to be detected at quote values above 1.30.

### In vitro splice assay using *EYS* minigenes

Two intronic *EYS* variants, c.2992_2992+6delinsTG and c.3877+1G>A were tested with pSPL3 exon trapping vector (Invitrogen, Carlsbad, CA) as described previously by Jonsson et al^43^. Genomic DNA from RP103 and RP15 patients was used for PCR amplification for fragments with *EYS* c.3877+1G>A and c.2992_2992+6delinsTG, respectively.

PCR was performed in 25 μl volume with 0.2 μM forward primer with EcoRI- and reverse primer with NotI at 5´-endsites (Supplementary Table S1) and 1.25U of Taq-DNA polymerase with 5′-3′ exonuclease activity (Ampliqon A/S, Odense, Denmark). Ligation of the amplicons was performed according to manufacturer´s instructions by adding 50 ng of pGEM-T Easy vector (Promega, Fitchburg, WI, USA), 3 μl of PCR product, 3U of T4 DNA ligase and 1X Rapid Ligation buffer to a final reaction volume of 10 μl. For propagation of the pGEM-T Easy vector, XL10-Gold ultracompetent cells (Stratagene, La Jolla, CA, USA) were transformed by adding the pGEM-T ligation reaction to 50 μl of XL10-Gold suspension as described elsewhere. Plasmid DNA was extracted from the overnight cultures (1 × LB with 100 µg/ml carbenicillin) using standard protocol. The DNA concentration was measured on a NanoDrop 1000 v.3.8.1 (Thermo Fisher Scientific, Waltham, MA, USA). To identify clones with correct inserts in the pGEM-T vector, 0.5–1 μg of each plasmid DNA was treated with 3U of EcoRI and NotI (New England Biolabs, Ipswich, MA, USA) for 1–2 h at 37 °C and visualized on a 1% agarose gel. Plasmids with correct size of insert were analysed by Sanger sequencing to identify wild type (wt) and mutant (mt) constructs and to rule out any sequence deviations generated by PCR. Sanger sequencing was performed using BigDye Terminator v3.1 Cycle Sequencing Kit (Applied Biosystems). For minigene construction, pSPL3 was digested with EcoRI and NotI and the wildtype and mutant inserts excised from pGEM-T Easy vector were ligated into the pSPL3 vector using the same ligation protocol as for pGEM-T Easy system followed by transformation into XL10-Gold ultracompetent cells. Ratios for pSPL3 versus inserts were calculated using an online tool at www.promega.com/a/apps/biomath/?calc=ratio. Sequence confirmation was done by Sanger sequencing with pSPL3 specific primers: forward SD6-5′- TCTGAGTCACCTGGACAACC and reverse SA2-5′- ATCTCAGTGGTATTTGTGAGC (Supplementary Information). Upon identification of one mutant and one wildtype minigene construct in the pSPL3 vector, the clones were propagated, and plasmid DNA was purified by midi-preparation using NucleoBond Xtra Midi kit (Macherey–Nagel, Düren, Germany).

Human Embryonic Kidney cells, HEK293T Lenti-X (Clontech Laboratories, Mountain View, CA, USA) and Human Retinal Pigmented Epithelium cells, ARPE-19 (ATCC CRL-2302, Manassas, VA, USA) were transfected with wt and mt pSPL3 minigenes, (*EYS* c.3877+1G>A and *EYS* c.2992_2992+6delinsTG). 2 × 10^5^ HEK293T or ARPE-19 cells were seeded in 24-well plates. HEK293T cells were cultures in DMEM medium (Gibco Laboratories, Gaithersburg, MD, USA) containing 1X Glutamax (Gibco) and ARPE-19 cells were grown in DMEM: F12 medium (Gibco). Both growth medium additionally contained 10% fetal bovine serum (Gibco) and 1X PenStrep (Gibco). The cells were transfected after 24 h using the Lipofectamine 3000 system (Thermo Fisher Scientific). All transfections were done twice with HEK293T and once with ARPE-19 cells. For each well, 1 μl Lipofectamine 3000 and 0.8 μg pSPL3 vector DNA was used. Total RNA was extracted 46–48 h after transfection with NucleoSpin RNA Plus (Macherey–Nagel) and reverse transcribed into cDNA in 20 µl reactions containing 100U Superscript III reverse transcriptase (Invitrogen, Carlsbad, CA, USA), 200 ng random hexamers (Thermo Fischer), 0.5 mM dNTP (Roche Diagnostics, Mannheim, Germany), 5 mM DTT (Invitrogen), 1 × First Strand Buffer (Invitrogen) and 128–1000 ng RNA. The cDNA reactions were incubated at 25 °C for 5 min, 50 °C for 60 min and 70 °C for 15 min. Thereafter, PCR was performed on 5 µl cDNA (estimated 32–100 ng) using pSPL3 specific primers SD6 and SA2 (Supplementary Table S1), 0.75U AmpliTaq Gold DNA Polymerase, (Applied Biosystems), 1 × PCR Buffer II (Applied Biosystems), 1.5 mM MgCl_2_ (Applied Biosystems) 0.2 mM dNTP (Roche Diagnostics) with a temperature profile of 95 °C for 12 min followed by 35 cycles of 95 °C for 30 s, 55 °C for 30 s and 72 °C for 30 s and a final extension at 72 °C. The PCR products were separated on 1% agarose gels and visualized under UV-light using ethidium bromide.

### Clinical evaluation

A full ophthalmic examination was performed at Eye Clinic of northern Sweden, and the clinical diagnosis was based on visual acuity, electroretinography (ERG) findings, fundus photography and Optical Coherence Tomography (OCT) visual fields.

#### Psychophysical methods and clinical examination

Visual acuity (VA) was measured using a LogMAR (Logarithm of the Minimum Angle of Resolution) chart. Slit-lamp examination, biomicroscopy and fundus examinations were performed, and fundus images were taken. Visual fields were measured with a Goldmann perimeter. Macular morphology and thickness were measured with optical coherence tomography (OCT), using a Topcon 3D OCT 2000 (Topcon Medical Systems, Oakland, NJ, USA). A pseudo-colour two-dimensional map of retinal thickness was used to confirm the retinal morphology.

#### Electrophysiological methods

Full-field electroretinograms (ERG) were recorded using Burian-Allens bipolar electrodes on an Espion Profile Ganzfeld ERG machine (Diagnosys LLC, Lowell, MA, USA) following the recommendations from the international society of Clinical Electrophysiological Vision^44,45^.

### Computational resources

For the interpretation of genomic variants, prediction of splicing effect and estimation of alternative splice junctions Alamut Visual v.2.9 (Interactive Biosoftware, Rouen, France) was used. To obtain splicing scores, the following software programs were applied: SpliceSiteFinder-like, Max- EntScan, NNSPLICE, Genesplicer, and Human Splice Finder. Frequencies of the variants present in controls were extracted from the Genome Aggregation Database (gnomAD) Version 2.1, which includes data of 125,748 exome sequences and 15,708 whole-genome sequences (https://exac.broadinstitute.org)^46^. We also used The Human Genomic Variant Search Engine, VarSome^47^. In this search engine information from more than 30 public databases is available with various annotations and predictions. Pathogenicity classification of sequence variants based on ACMG guidelines^20^ was automatically done in VarSome and controlled manually. According to the guidelines, variants can be classified as pathogenic, likely pathogenic, benign, likely benign, or uncertain significance using criteria for pathogenicity a) pathogenic, very strong (PVS) b) pathogenic, strong (PS); c) pathogenic, moderate (PM); and d) pathogenic, supporting (PP)^20^.

### Ethics approval

This study was approved by the Swedish Ethical Review Authority (Etikprövningsmyndigheten) (Dnr:2019–01744 dated 2nd April 2019).

### Consent to participate

Informed written consent was obtained from all participants of this study. For patient RP6795 written consent was obtained from her parents.

### Consent for publication

We confirm that we have obtained written permission to use data from all individuals included in this article.

## Supplementary Information

Supplementary Information.

## Data Availability

The authors declare that all data supporting the findings of this study are available within the article and its Supplementary Information files. The datasets generated, used, and analysed during this study are available from the corresponding author on reasonable request.
