# Supplementary material for: EYS mutations and implementation of minigene assay for variant classification in EYS-associated retinitis pigmentosa in northern Sweden
Source: Sci Rep. 2021 Apr 8;11:7696. doi: 10.1038/s41598-021-87224-9 (PMC8032658; doi:10.1038/s41598-021-87224-9)

Supplementary Information

EYS mutations and implementation of minigene assay for variant classification in EYS-associated retinitis pigmentosa in northern Sweden

Ida Maria Westin,[1] Frida Jonsson,[1] Lennart Österman,[1] Monica Holmberg,[1] Marie Burstedt,[2] Irina Golovleva[1, *]

[1]Medical Biosciences/Medical and Clinical Genetics, University of Umeå, 901 87 Umeå, Sweden
[2]Clinical Science/Ophthalmology, University of Umeå, 901 87, Umeå, Sweden
*Correspondence should be addressed to Dr. Irina Golovleva, Clinical Genetics, University Hospital of Umeå, 90185 Umeå, Sweden. ORCID 0000-0001-8741-0616. Telephone: +467856820; telefax: +4690128163; email: irina.golovleva@umu.se

Supplementary Figure S1

Pedigrees of the families in this study

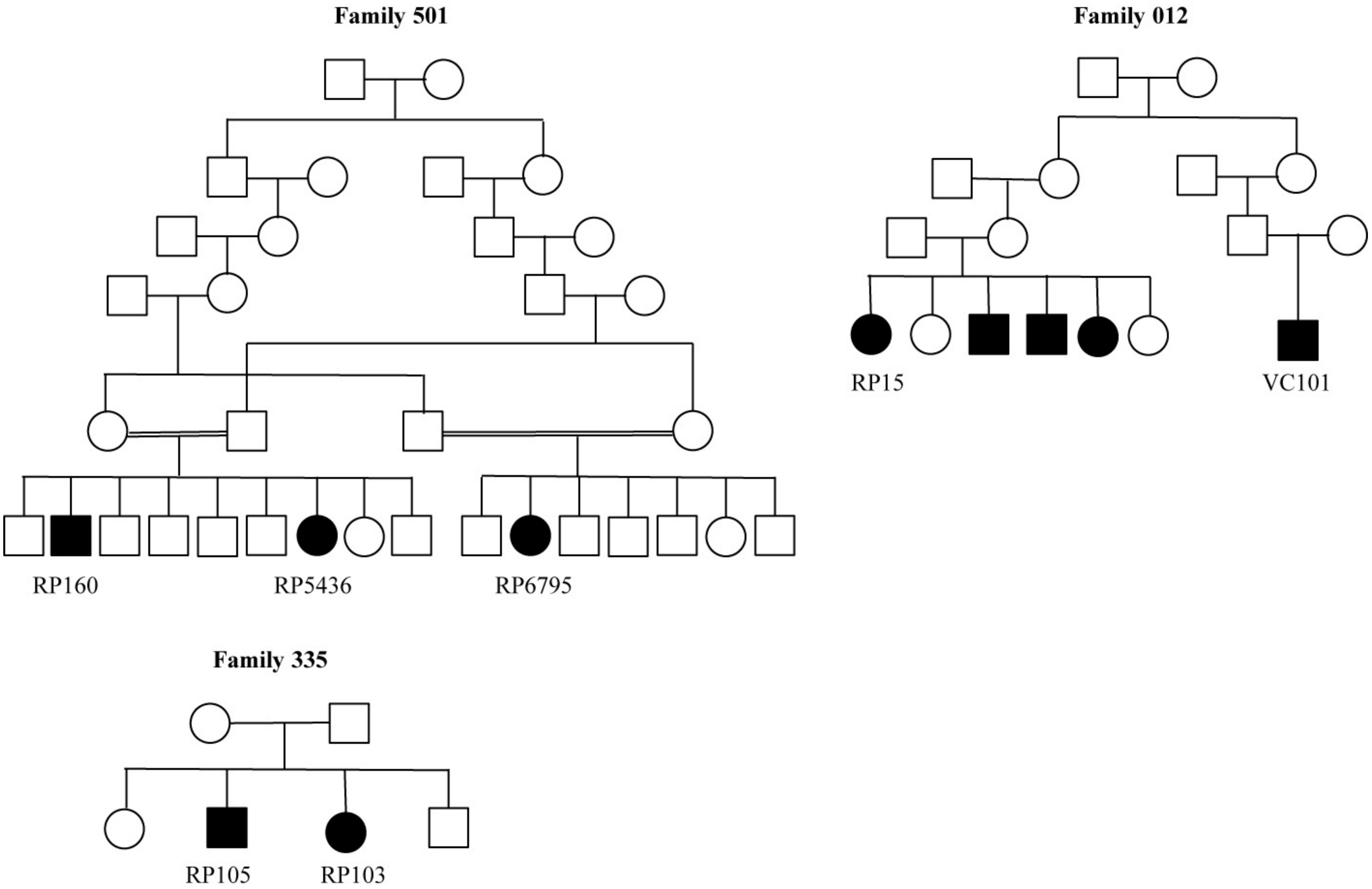

Circles and squares represent females and males, black and white symbols represent affected and unaffected, respectively; double lines - consanguineous marriage

Methods and outcome of cascade-targeted mutation analysis of *EYS* gene in arRP cohort from northern Sweden

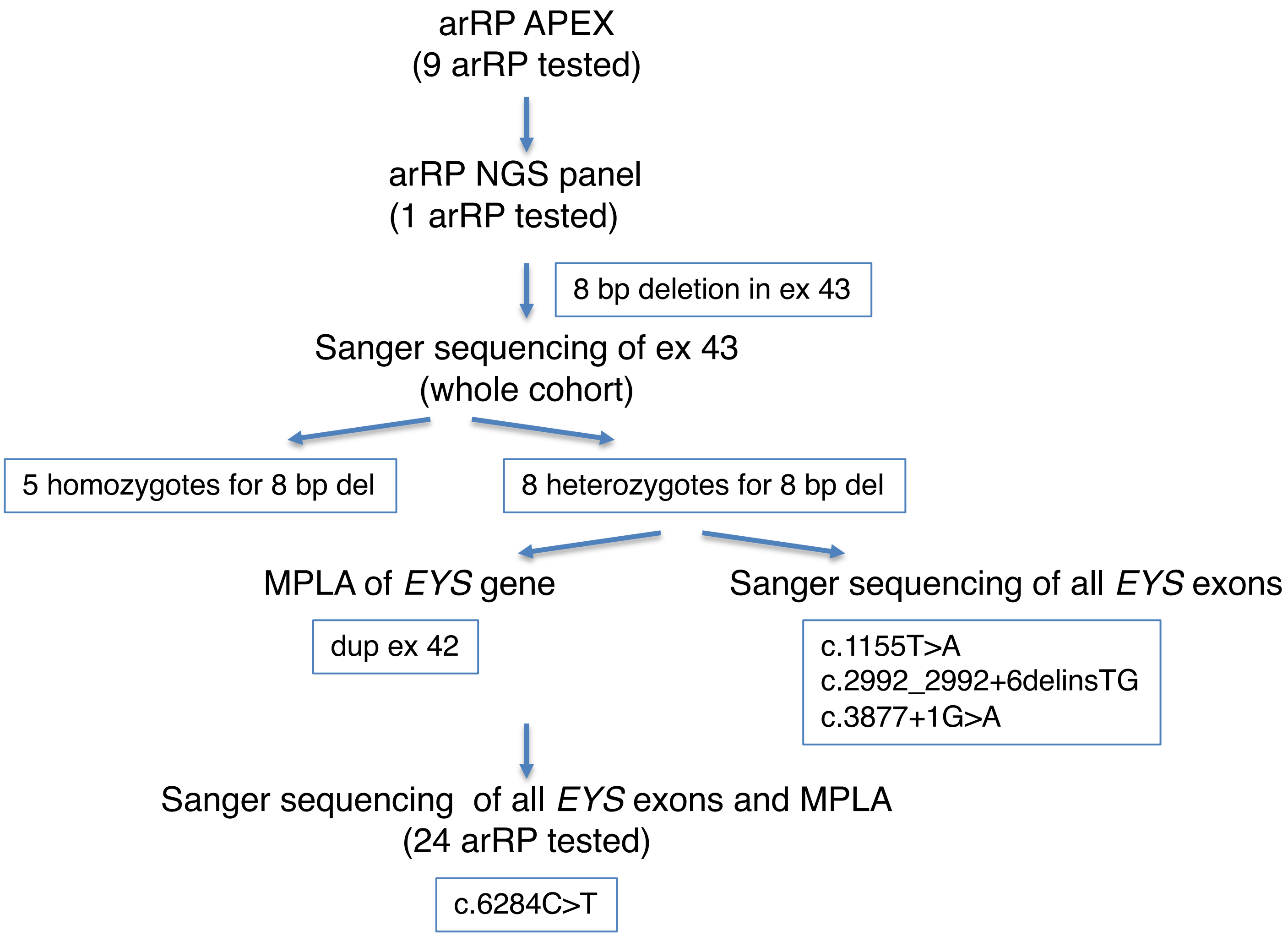

Our cohort (n=81) consisted of 65 arRP (3 probands, 4 family members and 58 simplex cases), 7 cases with either arRP or adRP and 9 adRP cases. Mutations detected at each step are shown framed.

EYS mutations in the cohort of patients of northern Sweden

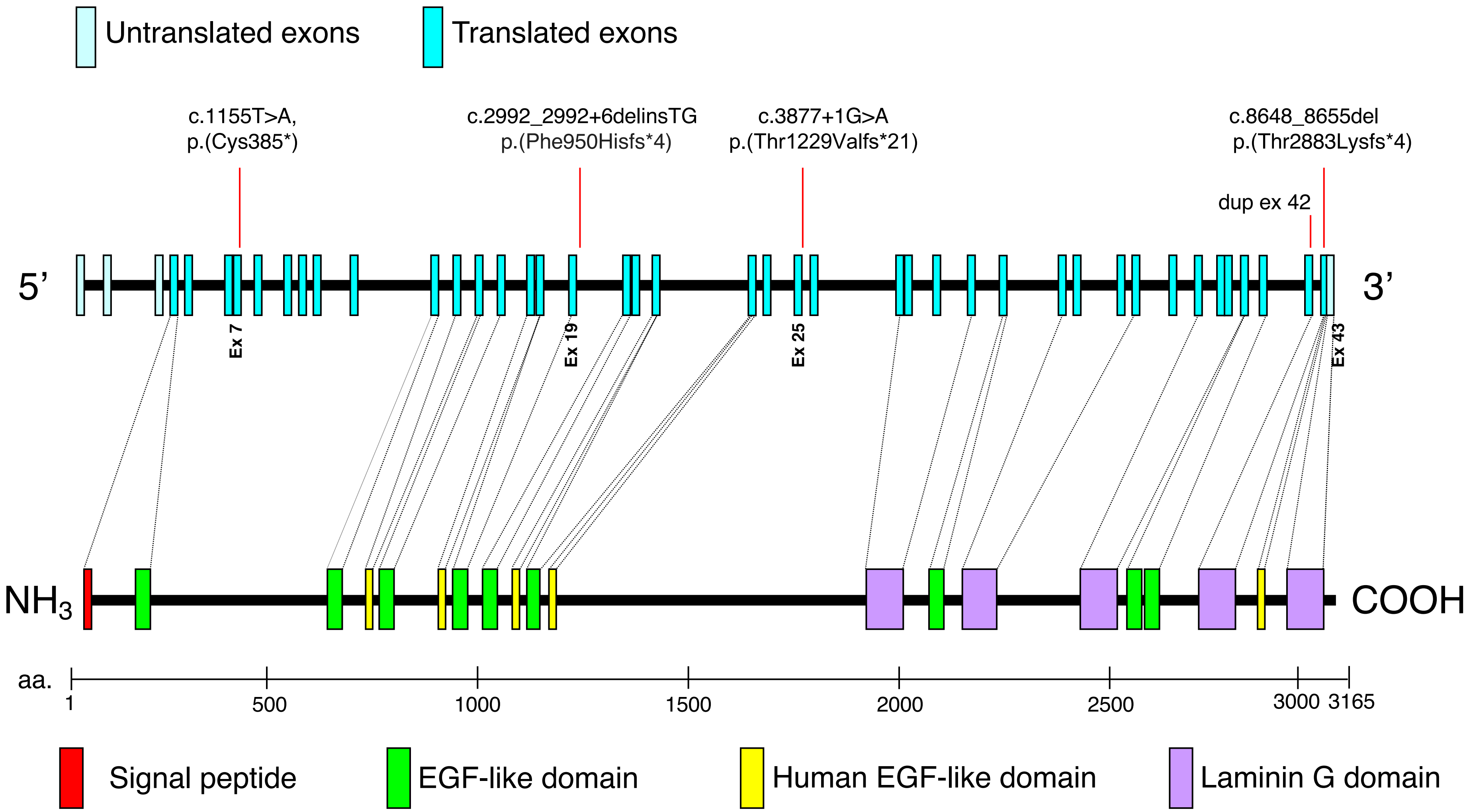

For protein source the following database was used. The Pfam protein families database: towards a more sustainable future by R.D. Finn, P. Coggill, R.Y. Eberhardt, S.R. Eddy, J. Mistry, A.L. Mitchell, S.C. Potter, M. Punta, M. Qureshi, A. Sangrador-Vegas, G.A. Salazar, J. Tate, A. Bateman. **Nucleic Acids Research** (2016) Database Issue 44:D279-D285

Supplementary Figure S4

MLPA analysis of *EYS* duplication

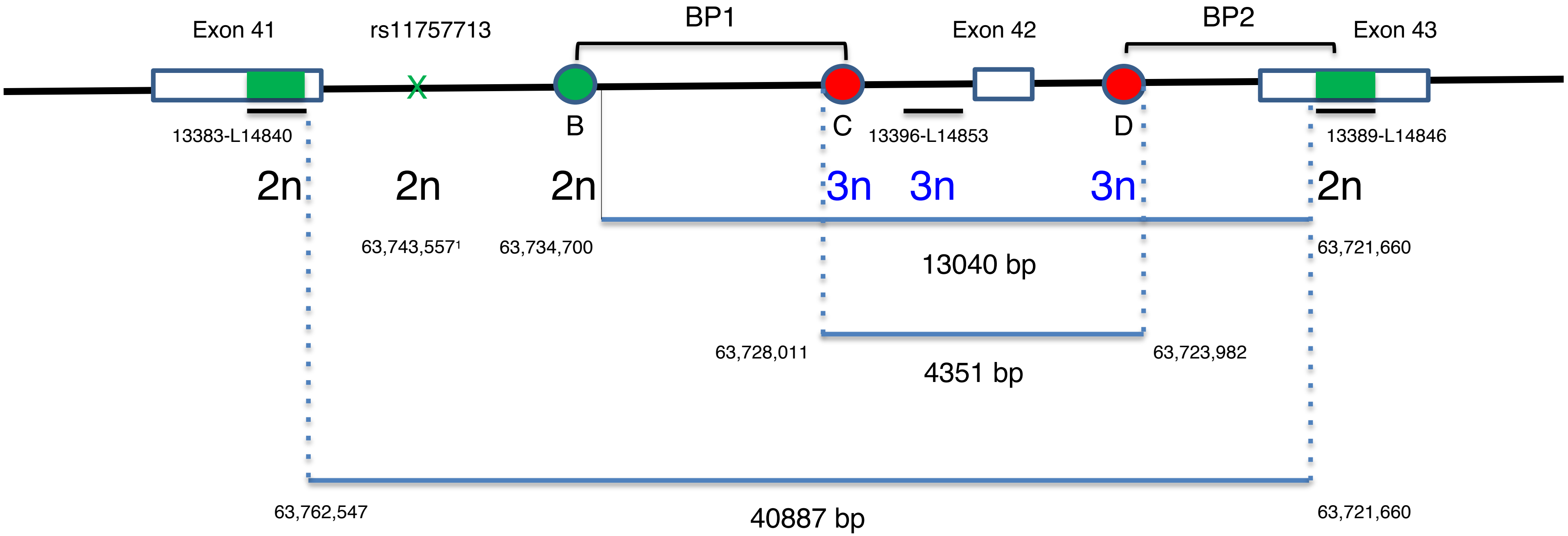

EYS exons are shown as open boxes. Cross is an informative SNP, rs 11757713. BP – breakpoints' regions of the duplication. MLPA probes from SALSA Kit P328-A2-0217 (MRC Holland, Amsterdam, Netherlands) are shown as black lines. B, C and D – MLPA probes designed in this study, their sequences are shown in the Table below. [1] – genomic positions in *EYS* sequence (NM_001142800.1) according to genome reference GRChr38/hg38. Two copies of MLPA probe is shown in green and denoted as 2n while duplication with three copies of MLPA probe is shown in red and described as 3n.

Probe	Sequence
Probe B-LHS<br>Probe B-RHS	5'-GGGTTCCCTAAGGGTTGGAGTCCTACCTGAGCTCCACATCTTCTCCAAAATACC<br>5´-TGCTTCTCCCACAGTCTTCCCCATTTCAGTTAATGATCTAGATTGGATCTTGCTGGCAC
Probe C-LHS<br>Probe C-RHS	5'-GGGTTCCCTAAGGGTTGGACAAGGAACCCCTATGTCAGAAACACCAGGAGGGTTTA<br>5´-TTAAAAATGGGGATGCTTTCTGAATCCCATCCCAGCATTCTAGATTGGATCTTGCTGGCAC
Probe D-LHS<br>Probe D-RHS	5'-GGGTTCCCTAAGGGTTGGAGGCCGAGGTGGGCGGATCACCTGAAGTCAGGAGGTCAAG<br>5´-ACCAGCCTGGCCAACATGGTAAAACCTCGCTCTACCCAAATCTAGATTGGATCTTGCTGGCAC

Primer sequences for PCR amplification of *EYS* exons for minigene constructs

EYS variant	Forward primer	Reverse primer	Expected size of splice products
c.3877+1G>A	5’-[1]AAATTTGGGCATGCAACTGT	5’-[2]TGAGGTCCAGTTGTCTATTCGA	453 bp
c.2992_2992+6delinsTG	5’-[1]CACACATAAAGAACATTTGAGCA	5’-[2]GCTCCCTGATGACTTTTGCC	406 bp
pSPL3	5’-TCTGAGTCACCTGGACAACC	5’-ATCTCAGTGGTATTTGTGAGC	260 bp (no insert)

[1]Forward primers have an EcoRI recognition sequence at the 5’end; [2]Reverse primers have a NotI recognition sequence at the 5’ end

Optical coherence tomography (OCT)

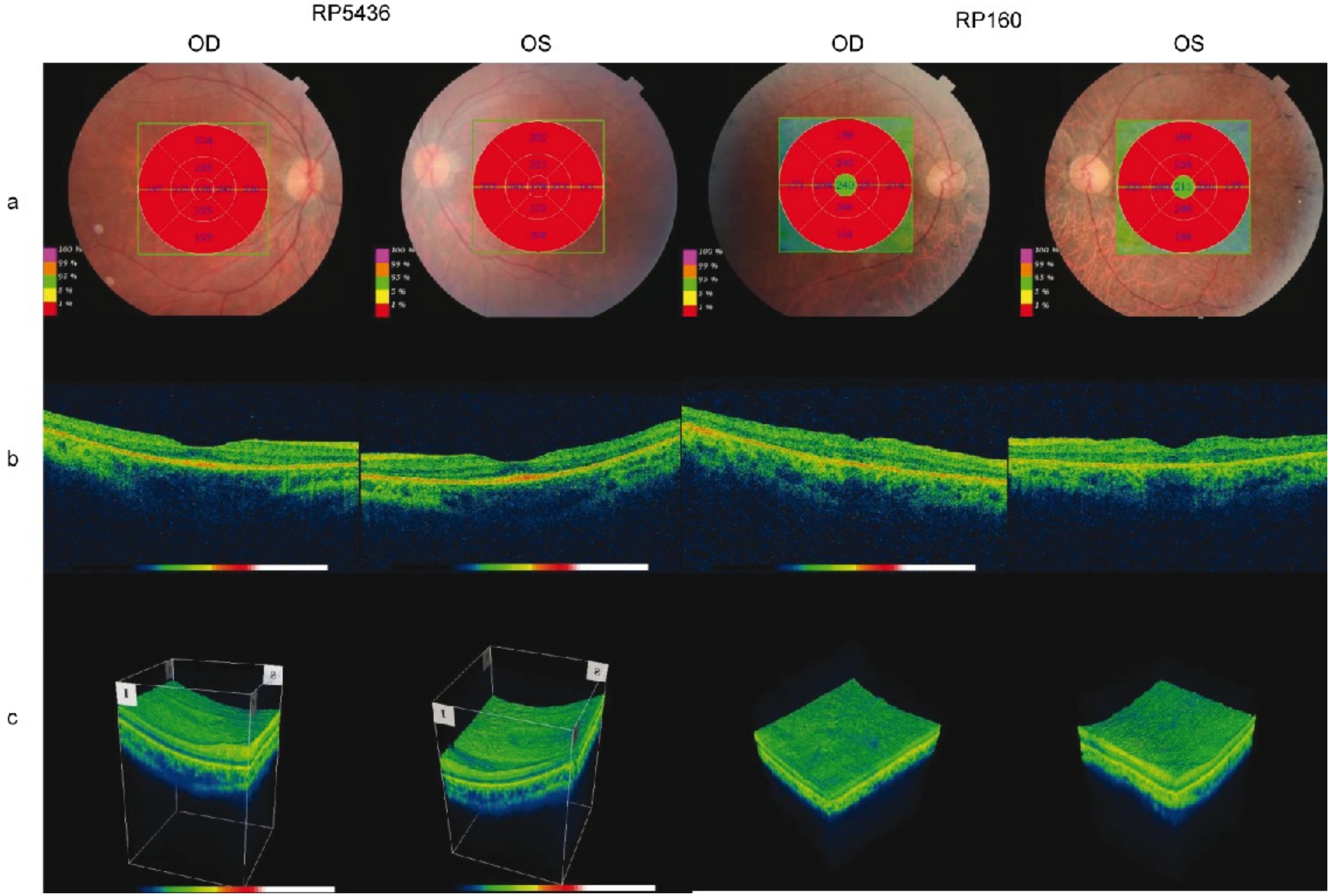

Optical coherence tomography (OCT) of two affected patients RP5436 examined at age of 18 yo and RP160 examined at age of 30 yo shows retinal thickness map (**a**), axial colour B-scan (**b**) and 3D scan (**c**).
I = inferior of the retina and S = superior of the retina

Full-field ERG of the three affected family members

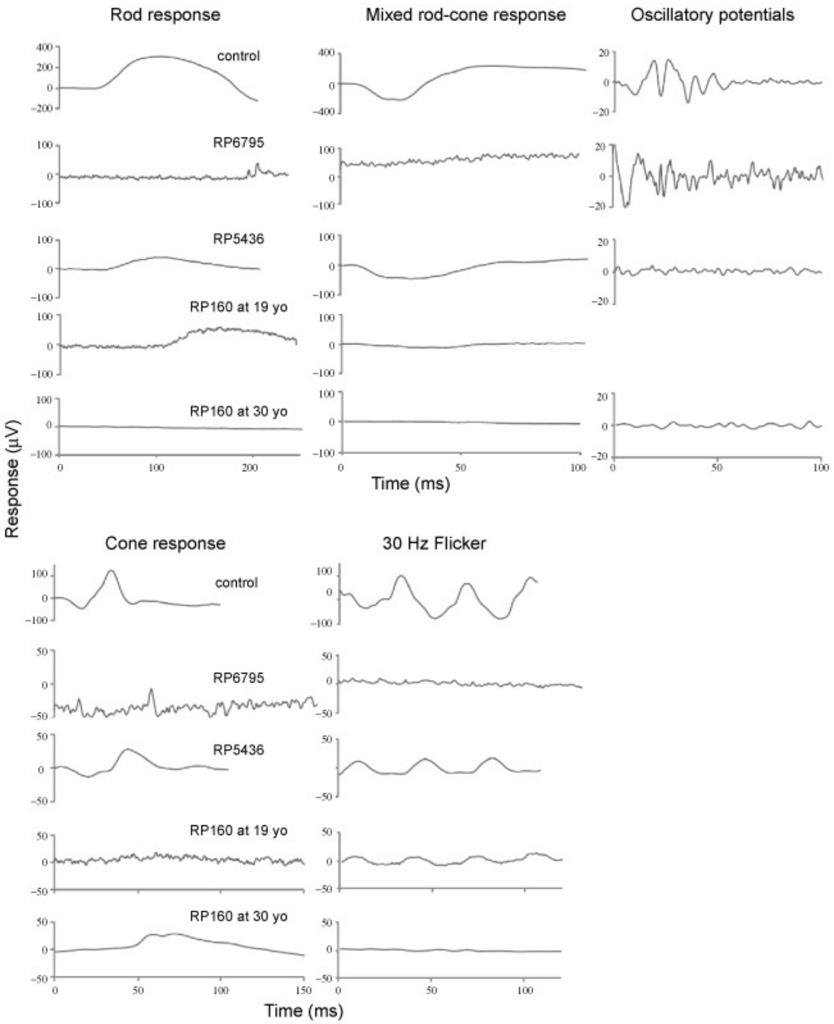

Case RP160 was examined twice at 19 and 30 years of age.
F = female; M = male. Details are described in the Results.

Supplementary Figure S7

Full-length gels of in vitro minigene splice assay – Fig.1b, Fig.1c, Fig 2b and Fig.2c

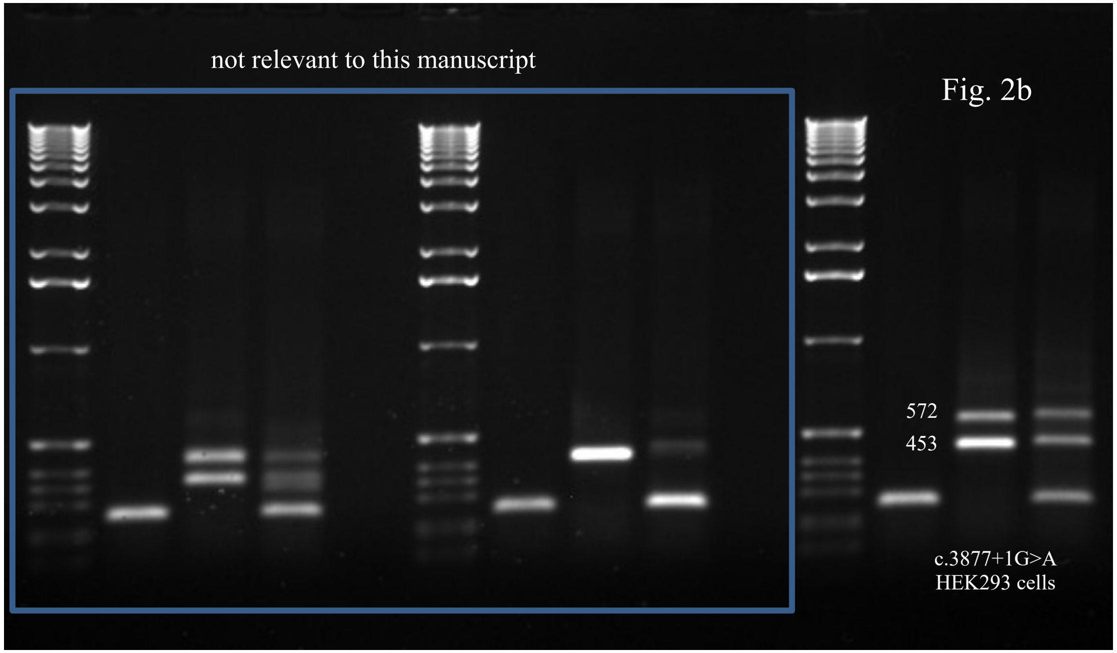

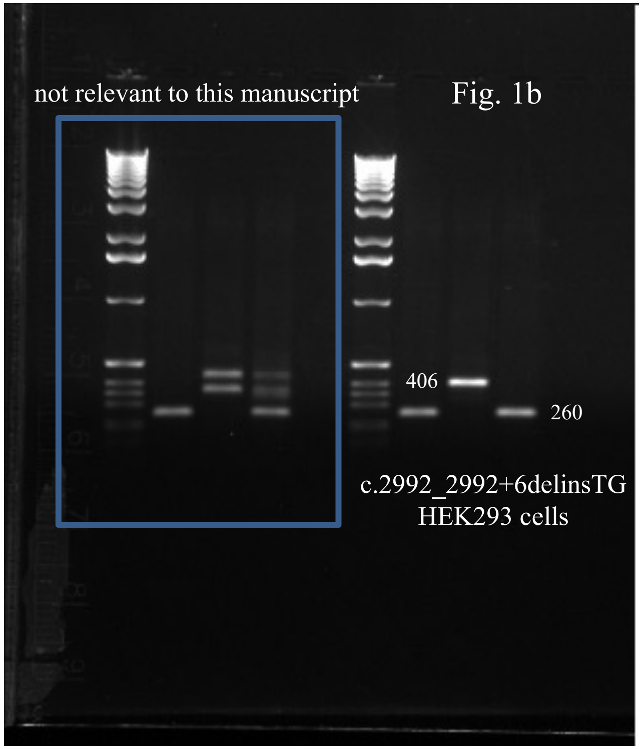

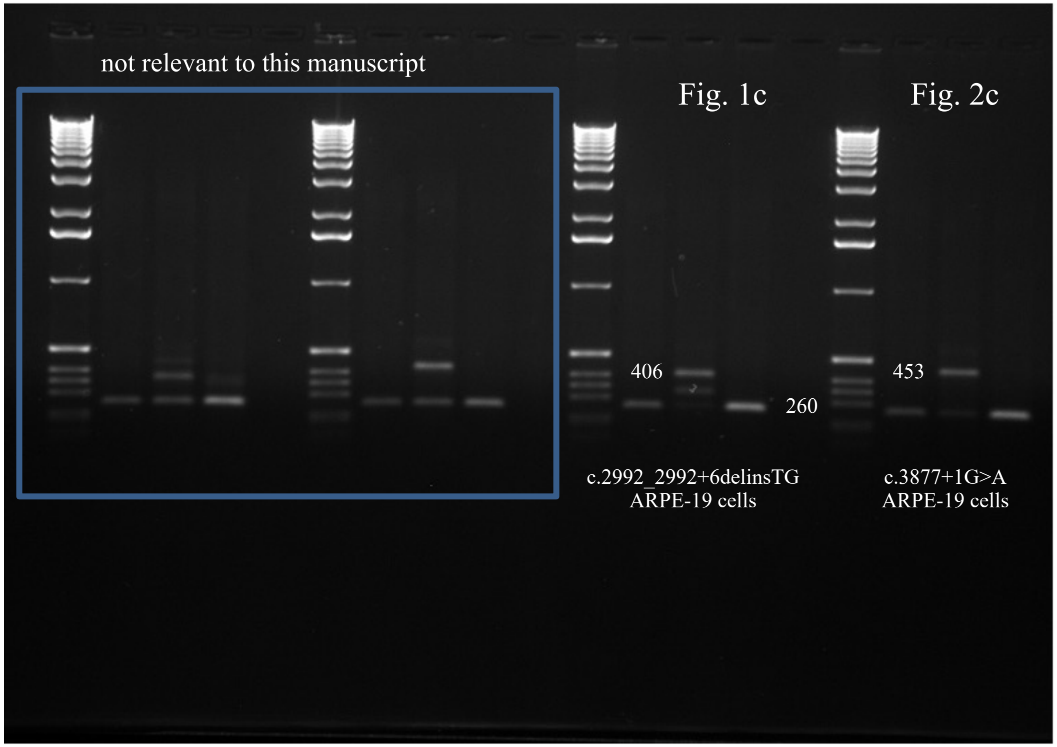

Supplement: Supplementary file 1 — Supplementary Information. [file 41598_2021_87224_MOESM1_ESM.pdf]
